# Plastid Genomes of Carnivorous Plants *Drosera rotundifolia* and *Nepenthes* × *ventrata* Reveal Evolutionary Patterns Resembling Those Observed in Parasitic Plants

**DOI:** 10.3390/ijms20174107

**Published:** 2019-08-22

**Authors:** Eugeny V. Gruzdev, Vitaly V. Kadnikov, Alexey V. Beletsky, Elena Z. Kochieva, Andrey V. Mardanov, Konstantin G. Skryabin, Nikolai V. Ravin

**Affiliations:** 1 Institute of Bioengineering, Research Center of Biotechnology of the Russian Academy of Sciences, 119071 Moscow, Russia; 2 Lomonosov Moscow State University, 119991 Moscow, Russia

**Keywords:** carnivorous plant, plastid genome, gene loss, RNA editing, Caryophyllales

## Abstract

Carnivorous plants have the ability to capture and digest small animals as a source of additional nutrients, which allows them to grow in nutrient-poor habitats. Here we report the complete sequences of the plastid genomes of two carnivorous plants of the order Caryophyllales, *Drosera rotundifolia* and *Nepenthes* × *ventrata*. The plastome of *D. rotundifolia* is repeat-rich and highly rearranged. It lacks NAD(P)H dehydrogenase genes, as well as *ycf1* and *ycf2* genes, and three essential tRNA genes. Intron losses are observed in some protein-coding and tRNA genes along with a pronounced reduction of RNA editing sites. Only six editing sites were identified by RNA-seq in *D. rotundifolia* plastid genome and at most conserved editing sites the conserved amino acids are already encoded at the DNA level. In contrast, the *N.* × *ventrata* plastome has a typical structure and gene content, except for pseudogenization of the *ccsA* gene. *N.* × *ventrata* and *D. rotundifolia* could represent different stages of evolution of the plastid genomes of carnivorous plants, resembling events observed in parasitic plants in the course of the switch from autotrophy to a heterotrophic lifestyle.

## 1. Introduction

Carnivorous plants are able to attract, catch, kill, and digest their prey, usually insects, and assimilate amino acids, peptides, and other nutrients resulting from digestion for growth [1,2]. The nutrients obtained are primarily used as a source of nitrogen and phosphorus, enabling these plants to survive in habitats with nutrient-poor soils and in oligotrophic aquatic environments. Nevertheless, all known carnivorous plants retain the ability to photosynthesize and to fix CO_2_, and they cannot grow heterotrophically with respect to organic carbon. About 700 species of carnivorous plants are currently recognized [3]. Carnivory evolved several times, with at least nine independent origins in five orders of angiosperms (Caryophyllales, Ericales, Lamiales, Oxalidales, and Poales) [4,5,6]. The majority of carnivorous plants belong to the orders Caryophyllales and Lamiales [3].

Another group of plants capable of receiving nutrients from other organisms is direct parasites and mycoheterotrophs, receiving nutrients, respectively, from host plants or through mycorrhizal fungi with which they associate. Contrary to carnivorous plants, parasitic plants depend on organic carbon derived from their hosts. Some parasitic plants retain photosynthetic capabilities, being hemiparasites, while others completely lose the ability to carry out photosynthesis [7]. Plastid genomes (plastomes) of parasitic plants represent model systems for studying the effects of relaxed selective pressure on photosynthetic function. In general, plastid genomes in angiosperms are highly conserved in structure, gene content, and gene order, but structural rearrangements and gene loss appear to be associated with the switch from autotrophy to heterotrophy [8,9,10]. Extensive studies of the plastid genomes of parasitic plants have revealed a reduction in size and gene content compared with their photosynthetic relatives, which correlates with the loss of genes encoding photosynthetic functions ([8,11,12], see [13] for a recent review). Such reduction could be minimal in hemiparasites and “early” parasites such as *Corallorhiza striata* [14] and *Cuscuta* sp. [15], extreme in some holoparasitic species [16,17,18,19,20], and even complete, as seen by the loss of the plastome in *Rafflesia lagascae* [21].

Contrary to parasitic plants, only several complete plastid genomes of carnivorous plants have been sequenced, and the possible effect of carnivorous lifestyle on the plastid genome remains poorly understood. Studies of plastid genomes of *Pinguicula ehlersiae*, three *Utricularia*, and seven *Genlisea* species as representatives of the three genera of the carnivorous family Lentibulariaceae, have revealed the loss and pseudogenization of the NAD(P)H dehydrogenase genes in some species as well as a significant increase of substitution rates and microstructural changes [22,23,24]. 

To get further insights into the molecular changes associated with the transition to carnivory, we sequenced the plastid genomes of the members of two other families of carnivorous plants, *Drosera rotundifolia* (Caryophyllales: Droseraceae) and *Nepenthes* × *ventrata* (Caryophyllales: Nepenthaceae). Phylogenetic age of both families is estimated in approximately 85 million years ago [3]. The family Droseraceae, comprising the genera *Drosera*, *Dionaea*, and *Aldrovanda* with more than 250 species, is the second most diverse carnivorous family after Lentibulariaceae [25]. *D. rotundifolia* is one of the most widespread sundew species, common in many areas of the Northern Hemisphere, including Europe, Siberia, and North America. *D. rotundifolia* typically thrives in wetlands such as bogs, marshes, and fens, but can also grow on open peatlands and wet sands. A typical plant has a size of about 3 to 5 cm. Leaves spread on the soil surface, with long petioles collected in a rosette. The edge and the upper surface of the leaf are covered with reddish glandular hairs in the form of heads on long stalks, which reach a length of 4–5 mm. The hairs secrete a sticky liquid in the form of shiny drops. They are sensitive to irritation, and when an insect hits a leaf, they bend and catch it.

Nepenthaceae comprises the single genus *Nepenthes*, which includes more than 160 species, most of which inhabit the tropical regions of South-East Asia [26]. *Nepenthes* use passive pitcher-shaped traps to catch small insects and are probably the most studied carnivorous plants [27]. *Nepenthes* × *ventrata* is a natural hybrid between *N. alata* and *N. ventricosa*, two species endemic to the Philippines [28]. This is a perennial medium-sized (about 30–50 cm high) plant with red cylindrical pitchers used to catch small insects. Pitchers of *Nepenthes* have been analyzed by RNA-seq and proteomics for the discovery of genetic traits related to carnivory [29,30].

Here we report the full plastome sequences of *D. rotundifolia* and *N.* × *ventrata* and present a comparative analysis of the plastomes of both species and their carnivorous and non-carnivorous relatives. The plastome of *D. rotundifolia* is the first one to be sequenced and released to the GenBank (KU168830) in the family Droseraceae. Recently, sequences of plastome of four other species of Droseraceae, *Drosera erythrorhiza* (GenBank KY651214), *Drosera regia* (KY679199), *Aldrovanda vesiculosa* (KY679200), and *Dionaea muscipula* (KY679201) have been published [31]. For the genus *Nepenthes*, the complete plastid genome sequences are known for *Nepenthes mirabilis* (MH286314, MK397879, MK397880, and MK397881), and a near-complete plastome sequence was reported for *Nepenthes graciliflora* (MH286314) [32,33]. We used these sequences for the comparative analysis. The results of the present study improve our understanding of the evolution of the plastid genomes of carnivorous plants and reveal some similar evolutionary patterns between carnivorous and parasitic plants.

## 2. Results

### 2.1. Plastome Size and Gene Content in D. rotundifolia

The plastid genome of *D. rotundifolia* is 192,912 bp in length and has a typical quadripartite structure with two single-copy regions and an inverted repeat. The increase in genome size compared with typical plastomes of flowering plants is primarily due to the extension of inverted repeats, which are 52,949 bp in length.

The *D. rotundifolia* plastome was predicted to contain 97 presumably intact unique genes (Table 1), which is somewhat less than its close non-carnivorous relative *Fagopyrum esculentum* (114 genes; [34]). Consistent with the ability to photosynthesize, the *D. rotundifolia* plastome contains a typical set of genes encoding photosynthesis-related proteins (photosystems I and II, cytochrome *b_6_f* complex, rubisco large subunit, and ATP synthase). The genes for plastid-encoded RNA polymerase and translation initiation factor InfA are also present. The most notable loss is the lack of all 11 genes coding for the NAD(P)H dehydrogenase complex (*ndhA–K*). Also missing are genes *ycf1* and *ycf2*, thought to be essential in plant plastids.

The functionality of the *accD* gene coding for the beta subunit of acetyl-CoA carboxylase is questionable because its predicted protein product contains an ~100 a.a. long N-terminal extension relative to typical AccD proteins (e.g., from *Nicotiana tabacum*). However, the whole functional domain (COG0777) is present, and the open reading frame remains non-interrupted. AccD protein, involved in fatty-acid synthesis and leaf development [35], is supposed to be essential for maintenance of the plastome in dicots [36].

The *clpP* gene, encoding the ATP-dependent proteolytic subunit of the Clp peptidase involved in protein metabolism within the plastid [36], is supposed to be essential and is present even in highly reduced plastomes of parasitic plants [16,20]. In the *D. rotundifolia* plastome, the only sequence corresponding to the N-terminal part of ClpP could be identified between genes *psbB* and *rpl20*, where the *clpP* gene is usually located in plastomes of angiosperms. However, it should be noted that accelerated evolution of ClpP was observed in several parasitic and photosynthetic lineages (see [17,37] for an example) to the extent that its sequence becomes difficult to identify.

The *D. rotundifolia* plastome encodes 27 tRNA species for all amino acids except alanine and lacks the tRNA genes *trnA-UGC*, *trnG-UCC*, and *trnV-UAC*, usually present in the plastomes of photosynthetic angiosperms. The loss of essential tRNA genes, including the above-mentioned ones, often occurs in the plastid genomes of parasitic plants [11,16,17,20] and it is assumed that the missing tRNA can be imported into the chloroplast from the cytosol.

All genes of ribosomal proteins typical of flowering plants were found in the plastome of *D. rotundifolia* (Table 1). An exception is the *rps18* gene, whose almost complete coding sequence (~100 a.a. residues) is part of the long open reading frame *orf641* capable of encoding a 641 a.a. protein. *Orf641* is located between genes *rpl33* and *rpl20*, which corresponds to the position of the *rps18* gene in typical chloroplast genomes [9]. The predicted protein product of *orf641* includes the N-terminal repeat-rich region followed by the almost complete Rps18 sequence, which has an identity of up to 65% with plastid Rps18 proteins. The N-terminal region includes 73 tandemly repeated sequences with GQKQPNI consensus (Figure S1). A GenBank search did not reveal close homologues of the N-terminal part of Orf641. The correctness of the assembly of the *orf641* sequence was confirmed via PCR and sequencing of the amplified fragment by the Sanger method.

Along with the loss of some conservative genes, a peculiar feature of the plastid genome of *D. rotundifolia* is the loss of introns in the remaining genes. Introns are present in *atpF*, *petB*, *petD*, *rpl16*, *rpoC1*, *ycf3* (two introns), *trnI-GAU*, and *trnL-UAA*, but appear to be lost in genes *clpP, rpl2*, and *rps16*. The *rps12* in the *D. rotundifolia* plastome is a trans-spliced gene as in most other angiosperms, but it consists of only two rather than three exons, indicating the lack of one (cis-spliced) intron. An unusual feature of the *D. rotundifolia* plastome is the loss of the intron in the *trnK* gene, which usually contains the *matK* gene coding for the maturase for splicing of group IIA introns [38]. The *matK* gene is preserved in the *D. rotundifolia* plastome and is located downstream of the intronless *trnK* gene. The presence of *matK* correlates with the retention of group IIA introns in *atpF* and *trnI-GAU* genes. The loss of the *trnK* gene and the retention of stand-alone *matK* has been described in different plant lineages [17,39,40], but the presence of intronless *trnK* is unusual.

### 2.2. Structural Rearrangements and Duplications in the D. rotundifolia Plastome

The gene order in chloroplast genomes of flowering plants is highly conserved, and its deviations are usually associated with movement of the boundaries of inverted repeats. However, in some lineages of flowering plants, for example cereals, geranium, and clover, and especially in parasitic plants, the order of genes is significantly different from the standard due to numerous genome rearrangements—translocations, duplications, inversions, and deletions [10,41,42].

**Figure 1:**
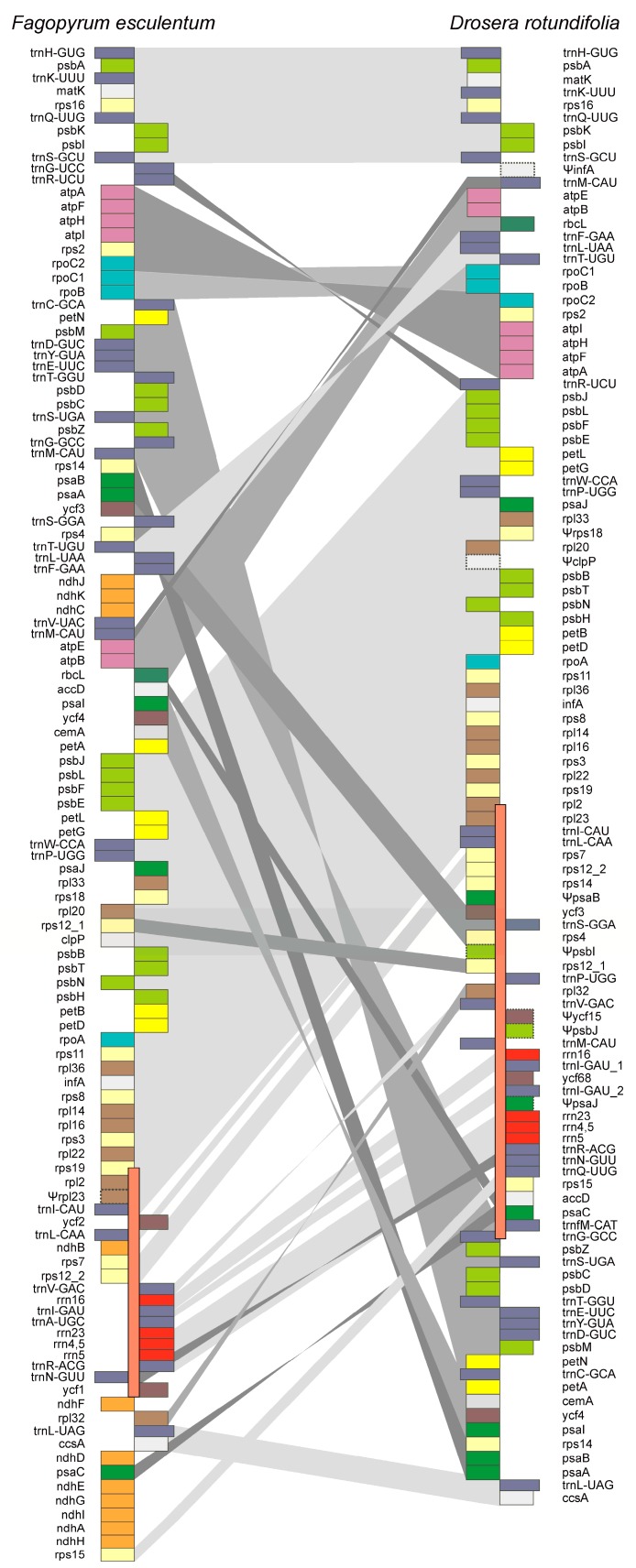
Schematic gene order comparison map between the plastid genomes of *D. rotundifolia* and *F. esculentum*. The linear representation of the circular mapping genomes using *trnH-GUG* genes as the starting point does not reflect the actual gene size or spacing between the coding regions. The inverted repeat is indicated by an orange rectangle on the main line; only one copy is shown. Boxes represent protein-encoding and RNA genes; genes transcribed in opposite directions are shown to the left and to the right of the main line. The gray areas between the genes highlight the relative locations of identical genes. Ψ denotes pseudogenes.

The comparison of the order of genes in the plastid genome of *D. rotundifolia* relative to the standard showed that the *D. rotundifolia* plastome, in addition to the above-mentioned deletions of particular genes, is characterized by large-scale rearrangements – inversion, translocation, and duplication of genes (Figure 1). Among these rearrangements are the transfer of *psaA-petN* genes from the large single-copy region (LSC) to the small one (SSC), insertion of the *petA-psaI* fragment into this cluster, inversion and translocation of *atpA-rpoC2* within the large single-copy region, etc. However, the plastome retains intact structures of the highly conserved S10 operon (*rps11*, *rpl36*, *infA*, *rps8*, *rpl14*, *rpl16*, *rps3*, *rpl22*, *rps19*, *rpl2*, *rpl23*) and the *rrn* gene cluster (*rrn16-rrn23-rrn4.5-rrn5*). These operons ensuring coordinated expression of the components required for assembly of the ribosome are conserved in the majority of chloroplast genomes.

It is likely that one of the main drivers of structural rearrangements and an increase in the size of the plastome was the duplication of extended fragments, accompanied by the insertion of their additional copies into other regions of the plastome. An analysis of the presence of repeats revealed that after excluding the large IR, the plastome of *D. rotundifolia* has many more total repetitive sequences than the plastomes of phylogenetically related species and other carnivorous plants. Repeat sequences account for about 23% of the *D. rotundifolia* plastome, while this value is less than 5% in the plastomes of other carnivores (Table 2). Increased repeat content is not associated with the accumulation of short tandem repeats often occurring in plastid genomes from slipped-strand mispairing [42,43], since their share in the *D. rotundifolia* plastome is not higher than in other plants (Table 2).

A high repeat content corroborates the presence of additional copies of some protein-coding and tRNA genes. Most of them are retained as truncated pseudogenes (*infA*, *psaB* (2×), *psbI* (2×), *psbJ* (2×), *psaJ* (2×)), but three duplicated genes remain intact and could be functional (*rps14*, *trnM-CAU*, and *trnP-UGG*).

### 2.3. The N. × ventrata Plastome Retains Conserved Structure and Gene Content

The plastid genome of *N.* × *ventrata* is 156,637 bp in length and includes a pair of inverted repeats of 25,190 bp separated by 19,208 bp-long and 87,049 bp-long single-copy regions. The predicted gene pool of the *N.* × *ventrata* plastid genome is typical for flowering plants and is almost identical to that of *F. esculentum* [34] except that the *rpl23* gene seems to be intact in *N.* × *ventrata*, as in most angiosperms [9]. The *N.* × *ventrata* plastome was predicted to contain 112 presumably intact unique genes (Table S1), including genes encoding photosynthesis-related functions, plastid-encoded RNA polymerase, and ribosomal proteins, as well as the conserved genes *infA*, *matK*, *cemA*, *clpP*, *accD*, *ycf1*, and *ycf2.* Unlike that of most carnivorous plants, the *N.* × *ventrata* plastome contains a complete set of genes coding for the NAD(P)H dehydrogenase complex (*ndhA–K*).

A total of 30 tRNA genes, seven of them having additional copies in the inverted repeats, were identified (Table S1). In contrast to *D. rotundifolia*, the set of tRNA genes in the *N.* × *ventrata* plastome is complete and can recognize all the codons present; therefore, no import of nuclear-encoded tRNAs is necessary. An analysis of the *N.* × *ventrata* plastome revealed the presence of all introns usually found in plastid genes and, as in most other angiosperms, the *rps12* gene is trans-spliced and consists of three exons.

The *N.* × *ventrata* plastid genome is colinear to those of tobacco [44] and *F. esculentum* [34] with respect to the gene order. No evidence of structural rearrangements or increased repeat content was found.

The only notable functional gene loss is the pseudogenization of the *ccsA* gene due to a 22 bp insertion approximately 507 bp downstream from the start codon, resulting in a frameshift. The mapping of transcriptome sequences obtained in RNA-seq experiments confirmed the transcription of the *ccsA* pseudogene and the presence of this insertion in the transcripts. It is unlikely that CcsA protein in plastids is replenished due to the *ccsA* copy which could be transferred to the nuclear genome, since the RNA-seq analysis did not reveal transcripts for CcsA-like proteins carrying a chloroplast transit peptide.

### 2.4. Identification and Prediction of RNA Editing Sites

RNA editing in the plastids of seed plants is a post-transcriptional modification that changes a cytosine (C) to a uracil (U) nucleotide, producing transcripts that are different from their DNA template [45]. RNA editing can alter the amino acid sequence of proteins and can also introduce new start and stop codons [45]. The average number of editing sites in non-parasitic higher land plants is around 30–40 [46], but some parasitic plants show a pronounced reduction in the number of editing sites [15]. To determine the editotypes of *D. rotundifolia* and *N.* × *ventrata*, we performed an *in silico* analysis for potential editing sites and identified them experimentally using RNA-seq analysis. The efficiency of editing was determined from the C versus U ratio at the respective position in the transcripts from the RNA-seq data.

Although our RNA sequencing approach based on polyadenylation-dependent sequencing library preparation was not suitable for quantitative analysis of plastid gene expression, a high average genome coverage by RNA-seq reads enabled the identification of the RNA editing sites. The RNA-seq analysis revealed 45 editing sites in 26 genes in the *N.* × *ventrata* plastome (Table S2). The most extensively edited appeared to be transcripts of genes *rpoC1* (5 sites) and *ndhD* (5 sites). 28 of these sites were also predicted by PREP-Cp software, indicating that the detected edits restored conserved amino acid residues in the corresponding proteins. Moreover, PREP-Cp predicted 37 additional sites not found in the RNA-seq data, most of which presumably remain unedited. Overall, the number and distribution of RNA editing sites in the *N.* × *ventrata* plastome are quite similar to those observed in other angiosperms [46], implying a lack of selection towards the loss of editing sites.

In contrast, only six RNA editing sites were identified by the RNA-seq analysis in the *D. rotundifolia* plastid genome (Table 3). Five of them were also predicted by PREP-Cp and matched conserved editing sites in genes *atpF*, *rps2* (2 sites), *rps14*, and *rpl23* found in other angiosperms [46]. One more site at codon position 73 in *rpl20* could convert serine to leucine, although this position is not conserved in Rpl20. An additional 31 sites were predicted by PREP-Cp but not confirmed by RNA-seq (Table S3).

## 3. Discussion

### 3.1. Evolution of the rps18 Gene in D. rotundifolia Plastome

**Figure 2:**
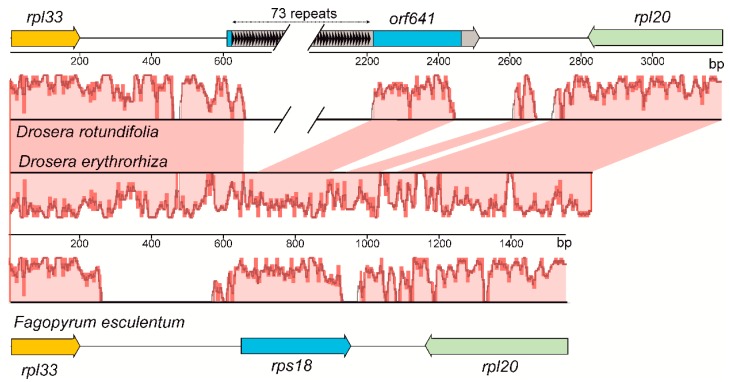
Comparison of the plastid genome regions comprising genes *rpl33*, *rps18*, and *rpl20* for *D. rotundifolia*, *D. erythrorhiza*, and *F. esculentum*. Regions between the start of *rpl33* and the start of the counter-oriented *rpl20* are shown; coordinate numbering for each plastome starts from the beginning of gene *rpl33*. Genes are indicated by rectangles with arrowheads. Black triangles within the *orf641* rectangle indicate 73 copies of the 21 bp-long repeat sequence coding for a peptide with GQKQPNI consensus in *D. rotundifolia*. The part of *orf641* that corresponds to the *rps18* sequence is indicated in blue. The central part shows alignment in Mauve; pink areas between *D. rotundifolia* and *D. erythrorhiza* sequences highlight homologous regions. Note that the Mauve profile obtained for *D. erythrorhiza* has been turned upside down for clarity of presentation.

A peculiar feature of the plastid genome of *D. rotundifolia* is the presence of a long *orf641*, predicted to encode a protein comprising an N-terminal repeat-rich region linked to a near-complete Rps18-like sequence. Such a gene has not been found in any other plastid genomes, including the recently sequenced plastomes of four other species of Droseraceae, *Drosera regia*, *Drosera erythrorhiza*, *Aldrovanda vesiculosa*, and *Dionaea muscipula* [31]. The appearance of *orf641* is an evolutionarily recent event and is likely the result of the insertion of the repeat-rich region (73 tandem repeats) at the 5′ end of the parental *rps18* gene. A comparison of the regions of plastid genomes comprising *orf641* and its flanking genes, *rpl33* and *rpl20*, revealed that the repeat-rich part of *orf641* is surrounded by sequences conserved in related species, including *F. esculentum* (Figure 2). 

We propose two possible explanations for the origin of the *orf641* in *D. rotundifolia*. One possibility is that the repeat-rich part of *orf641* was derived from lateral transfer from another organism. There is abundant evidence of horizontal gene transfer to plant mitochondrial and nuclear genomes [47], which is especially frequent in parasitic lineages [48]. However, to the best of our knowledge, gene transfer from the nuclear genome to the plastid has not been reported for land plants. Alternatively, the *orf641* gene in *D. rotundifolia* could result from recent multiple tandem duplication of repeats in the 5′-terminal region of the parental *rps18* gene. An analysis of nucleotide sequences of *rps18* genes revealed that accumulation of approximately 21 bp-long repeats in this region occurred in many species of angiosperms (especially in Poaceae and Campanulaceae), although the number of repeats did not exceeded 15 (Table S4). In some species repeats are present also in the 3′-terminal region of *rps18*. Both explanations leave open the question of the functionality of the Rps18-like part of Orf641. Preservation of the reading frame within the entire gene despite the presence of multiple repeats indicates its functionality, although the presence of a large N-terminal insert in Rps18 may be incompatible with the structure of the ribosome. It is possible that the formation of a protein close to the native Rps18 is possible due to the use of internal start codons within *orf641.*

### 3.2. Gene Loss and Genome Rearrangements in the Plastids in Three Families of Carnivorous Plants

Although the set of genes present in the plastid genomes of higher plants is highly conserved, there are numerous cases of the loss of some genes in different lineages. Most frequently lost is the *infA* gene encoding a translation initiation factor, the loss of which has been described in at least 24 lineages of angiosperms [49]. Examples of the loss of genes of individual ribosomal proteins are also known [50,51,52,53]. In some cases, it has been shown that loss of the chloroplast gene is accompanied by its transfer to the nuclear genome, and the lost protein is replaced by the one synthesized from the nuclear copy due to its acquisition of a signal sequence for transport into chloroplasts [49,52]. Of all the ribosomal proteins genes, only *rps18* was likely pseudogenized in the *D. rotundifolia* plastome, and such a mechanism could deliver an intact Rps18 to chloroplasts. However, the RNA-seq analysis did not reveal transcripts for Rps18-like proteins carrying a chloroplast transit peptide.

However, gene losses in *D. rotundifolia* are much more extensive and resemble events occurring in plastid genomes of parasitic plants. In the course of transition to a heterotrophic lifestyle NAD(P)H dehydrogenase genes are lost first, followed by genes responsible for photosynthesis (*psa*, *psb*, *pet*, *rbcL*, etc.), the plastid-encoded RNA polymerase, and ATP synthase genes [13,14]. The last to be lost are genes encoding ribosomal and tRNAs, ribosomal proteins, and some other essential genes (*accD*, *clpP*, *matK*, *ycf1*, *ycf2*). Gene losses are accompanied by a decrease in the size of the plastome, loss of introns, genomic rearrangements, and loss of the characteristic quadripartite structure. The analysis of the plastid genomes of *D. rotundifolia* and *N.* × *ventrata* revealed that they have common features with plastid genomes of parasitic plants at different stages of such a process. At the same time, obligate dependence of carnivorous plants on photosynthesis determines preservation of the complete set of photosynthesis genes, as well as the plastid-encoded RNA polymerase, and ATP synthase genes.

Partial or complete loss of 11 genes coding for subunits of the thylakoid NAD(P)H dehydrogenase (NADH) complex from the plastome has been reported for parasitic plants [17,54,55] and some fully autotrophic lineages (e.g., Pinaceae; [56]). The NAD(P)H dehydrogenase complex mediates electron cycling around photosystem I and balances the ratio of ATP and NAD(P)H. The latter is used for carbon fixation in the Calvin cycle [57]. NAD(P)H dehydrogenase is not absolutely required under normal conditions, but is important in stress conditions, such as increased or decreased light intensity, and low CO_2_ concentrations [58,59]. The loss of NAD(P)H dehydrogenase has been reported in carnivorous Lentibulariaceae [22,23,24]. In *Genlisea margaretae*, the genes *ndhC*, *D*, *F*, *G*, *H*, *J*, and *K* have been lost from the plastome, *ndhA*, *B*, *E*, and *I* remain as pseudogenes, and *ndhB* is intact [22]. The plastome of *Pinguicula ehlersiae* contains intact *ndhB*, *ndhA*, *D*, *E*, *G*, *H*, *I*, *J*, and *K* pseudogenes and completely lacks *ndhC* and *F* [22]. A different pattern was found in *Utricularia reniformis*, where the genes *ndhC*, *F*, *J*, and *K* have been lost from the plastome, and the genes *ndhA*, *B*, *D*, *E*, *G*, *H*, and *I* reside as truncated pseudogenes [23]. A complete set of *ndh* genes was found in the plastomes of *Utricularia macrorhiza* and *Utricularia gibba* [22,60]. It is likely that the reduced dependence of sundew, some other carnivorous plants on active photosynthesis, and a relatively stable habitat enable the loss of NAD(P)H dehydrogenase.

An interesting finding is the loss of the *ycf1* and *ycf2* genes in *D. rotundifolia*, not reported in the plastids of carnivorous plants of the family Lentibulariaceae. In contrast to *ndh* genes, *ycf1* and *ycf2* are essential when tested by gene knockout in tobacco [61], but losses of these genes have been reported in plant plastids [9,42] including some parasitic plants [18]. It was proposed that Ycf1 is involved in protein import into chloroplasts [62], but this proposal was questioned later [63].

Plastomes of three *Nepenthes* species, *N.* × *ventrata*, *N. graciliflora* (GenBank MH286314) and *N. mirabilis* (GenBank MH346374, MK397881, and MK397880), have retained a standard structure and a set of genes typical for photosynthetic flowering plants. A notable exception is the *ccsA,* which is a pseudogene in *N.* × *ventrata* due to a 22 bp insertion. GenBank searches showed that similar frameshifting insertions are present in the *ccsA* genes of *N. graciliflora* and *N. mirabilis*, indicating that the pseudogenization of *ccsA* could be a common trait in *Nepenthes*. The *ccsA* gene codes for a cytochrome C biogenesis protein and is conserved among photosynthetic plants [9], but it is lost along with other photosynthesis-related genes in achlorophyllous parasitic plants [10,16,19]. The only other known example of the functional loss of *ccsA* in photosynthetic plants is its pseudogenization in an obligate hemiparasitic species, *Viscum album* (Santalales: Viscaceae) [64]. Therefore, this loss could be enabled by a reduced dependency of hemiparasitic and carnivorous plants on active photosynthesis.

Changes in the plastid genome of *D. rotundifolia* resemble those described for hemiparasitic plants—the loss of not only NAD(P)H dehydrogenase but also some other conservative genes, loss of introns, accumulation of repeats, and multiple rearrangements of the genome. However, the photosynthetic apparatus and the functions associated with its operation are fully preserved. A similar pattern of plastome structure and gene content was recently described for four other Droseraceae species, *D. erythrorhiza*, *D. regia*, *A. vesiculosa*, and *D. muscipula* [31]. All four plastomes lacked all *ndh* genes. The plastome of *D. erythrorhiza* also lacks intact *ycf1, ycf2, psbK, rpl23, rps16,* and possibly *rpl32*. Losses of protein-coding genes besides *ndh* in the plastid genomes of three other species were not reported [31]. As in the case of *D. rotundifolia,* the losses of essential tRNA genes have been reported, namely *trnG-UCC* and *trnV-UAC* in *D. muscipula, trnA-UGC*, *trnG-UCC*, *trnI-GAU*, and *trnV-GAC* in *D. erythrorhiza.* Plastomes of Droseraceae differ significantly in size, from 117,589 bp in *D. muscipula* to 192,912 bp in *D. rotundifolia* (Table 2), mostly due to variation in the size of IR region. The comparison of the order of genes in the plastomes of Droseraceae relative to the standard for angiosperms revealed that all plastomes experienced multiple structural rearrangements, including inversions, translocations, and duplications [31]. The order of genes in all Droseraceae plastomes is different, with the largest number of rearrangements observed in *D. rotundifolia*. This is consistent with the observation that the *D. rotundifolia* plastome contains the largest number of repeats compared to other plastomes of Droseraceae (Table 2).

### 3.3. Reduction of RNA Editing Sites in Plastomes of Some Carnivorous Plants

A notable feature of the *D. rotundifolia* plastid genome, absent in *N.* × *ventrata*, is the reduction of the number of RNA editing sites. Only six sites were identified by RNA-seq, contrary to the 30–40 editing sites typically found in non-parasitic flowering plants. Even considering the loss of *ndh* genes containing up to half of the editing sites of angiosperm plastid transcripts, the number of sites in the remaining genes is unusually low and they were found only in *atpF*, *rps2*, *rps14*, *rpl20*, and *rpl23*, but not in the usually heavily edited genes *rpoA*, *rpoB*, *rpoC1*, *rpoC2*, *accD*, and *matK*. At most conserved editing sites, the conserved amino acids in *D. rotundifolia* are already encoded at the DNA level, which makes editing superfluous. Interestingly, only 7 editing sites were identified by RNA-seq in the plastome of another carnivorous plant, *U. reniformis* [23]. Previously, a pronounced reduction of editing sites and a reduction of editing efficiency were found in some parasitic plants [15]. Since RNA editing is a mechanism of post-transcriptional regulation of gene expression in the chloroplast, its reduction in the plastids of parasitic and carnivorous plants could be associated with a decrease in their dependence on active photosynthesis.

### 3.4. Convergent Plastid Genome Evolution in Carnivorous and Parasitic Plants

Overall, this study revealed the remarkable similarities between the plastid genomes of carnivorous and parasitic plants. Such convergence between carnivorous and photosynthetic parasitic plants was noted by Wicke et al. [22], in which a loss of NAD(P)H dehydrogenase genes and a significant relaxation of purifying selection in ATP synthase complex, photosystem I, and in several other photosynthesis and metabolic genes were observed in three genera of the carnivorous family Lentibulariaceae. However, in terms of structure, besides the losses of the *ndh* genes, plastomes of these species are collinear in gene order to those in the majority of angiosperms and show no structural rearrangements. 

Plastid genomes of the members of the family Droseraceae and especially of *D. rotundifolia*, revealed more pronounced features typical for parasitic plants such as the loss of some housekeeping genes, the loss of introns and RNA editing sites, accumulation of repeats, and structural rearrangements of plastomes. It can be proposed that the reason for this convergence may be the possibility of obtaining nutrients from other organisms, common to carnivorous and parasitic plants. Even though all carnivorous plants have an obligatory dependence on photosynthesis and primarily use prey as a source of nitrogen, direct uptake of organic carbon from prey has been reported [2,65]. Moreover, feeding on prey increases photosynthetic efficiency in *Drosera capensis* [66]. The outcome of such lifestyle could be the relaxation of purifying selection in photosynthesis and photosynthesis-related genes further promoting gene loss and structural instability of the plastome, as it occurs in parasitic plant species.

## 4. Materials and Methods 

### 4.1. Plant Material and DNA Isolation

Plant material of *D. rotundifolia* was collected from a wetland in the Moscow region, Russia. *N.* × *ventrata* plants were grown in the greenhouse of Research Center of Biotechnology RAS, Moscow, Russia. The voucher specimens were deposited in the herbarium of the Institute of Bioengineering, Research Center of Biotechnology RAS (accession numbers DRT-CB1 for *D. rotundifolia* and NEP-CB1 for *N.* × *ventrata*). The same *N.* × *ventrata* plant was previously used for the sequencing of the mitochondrial genome [67]. The leaves of several *D. rotundifolia* plants and leaves of a single *N.* × *ventrata* plant were used for the extraction of total genomic DNA using a CTAB-NaCl method [68].

### 4.2. Sequencing and Assembly of the Plastid Genome of D. rotundifolia

Total genomic DNA of *D. rotundifolia* was sequenced with a Roche GS FLX Genome Sequencer (Roche, Basel, Switzerland) using the Titanium XL+ protocol for a shotgun genome library. About 81 Mb of cleaned sequences with an average read length of 609 nt was generated. *De novo* assembly was performed with Newbler Assembler v. 2.9 (454 Life Sciences, Branford, CT, USA) with default settings, which yielded eight long chloroplast DNA contigs with 36-fold average coverage. These contigs were identified based on sequence similarity to chloroplast genomes of angiosperms and high coverage. The complete plastid genome sequence was obtained upon the generation of appropriate PCR fragments covering the gaps between the contigs and their sequencing by the Sanger method on an ABI PRISM 3730 analyzer (Applied Biosystems, Foster City, CA, USA). The list of primers is available in Table S5. To verify the correct assembly of the reconstructed plastid genome, raw reads were mapped against the obtained sequence with GS Reference Mapper (454 Life Sciences).

### 4.3. Sequencing and Assembly of the Plastid Genome of N. × ventrata

The plastid genome of *N.* × *ventrata* was sequenced using the Illumina technique. The sequencing of a TrueSeq DNA library on an Illumina HiSeq 2500 system (Illumina, San Diego, CA, USA) generated 3 million single-end reads with a length of 250 nt. Primer and quality trimming was performed with Cutadapt v. 1.17 [69] and Sickle v. 1.33 (https://github.com/najoshi/sickle), respectively. Cutadapt was used with default settings, and Q33 score was used for Sickle. The reads were *de novo* assembled using SPAdes v. 3.7.1 [70]. Fifteen contigs representing the plastid genome were identified based on sequence similarity to chloroplast genomes of angiosperms and high average coverage (98×). Contigs were joined to produce a single circular molecule using the Bandage v. 0.8.0 tool [71]. Reads spanning junctions between the single copy regions and inverted repeats were used to infer joins at these sites. The correctness of the assembly of the complete circular plastid genome was verified by mapping Illumina reads back to the assembled sequence using Bowtie 2 [72], and no evidence of misassembly was found.

The sequences of the plastid genomes of *D. rotundifolia* and *N.* × *ventrata* were submitted to GenBank under accession numbers KU168830 and MK758110, respectively.

### 4.4. Plastid Genome Annotation and Analysis Tools

Plastid genome annotation was performed using DOGMA [73], with further manual correction using similarity searches against previously annotated plastid genomes. Repetitive sequences were identified by comparing each genome to itself with NCBI BLASTN+ v. 2.2.24 (MEGABLAST), using a word size of 7 and an E-value threshold of 1 × 10^−6^ [74]. In addition, short tandem repeats were identified with Phobos v. 3.3.12 (http://www.ruhr-unibochum.de/ecoevo/cm/cm_phobos.htm). This analysis was restricted to sequences of at least 20 bp in length, containing two or more copies of a perfect repeating unit from 2 to 40 bp in length. One copy of the large IR was removed from each genome prior to repeat analyses. Alignment of plastid genome regions of *D. rotundifolia*, *D. erythrorhiza*, and *F. esculentum* comprising genes *rpl33*, *rps18*, and *rpl20* was performed using the program Mauve [75], with default settings. 

### 4.5. RNA Editing Analysis

The leaves of several *D. rotundifolia* plants were collected for transcriptome analysis and pooled. Total RNA was isolated from approximately 300 mg of tissue using an RNeasy Plant Mini kit (Qiagen, Valencia, CA, USA). mRNA library preparation was performed using an NEBNext^®^ mRNA Library Prep Reagent Set for Illumina^®^ according to the manufacturer’s instructions (New England BioLabs, Ipswich, MA, USA). The library was sequenced on Illumina MiSeq according to the manufacturer’s instructions, generating 24,629,238 paired-end reads (2 × 250 bp). A total of 20,964,026 high-quality read pairs were filtered after removal of adapter sequences and quality trimming with Cutadapt v. 1.17 [69] and Sickle v. 1.33 (https://github.com/najoshi/sickle), respectively. RNA-seq read data has been deposited in the NCBI SRA database under accession SRR8948654.

Leaves and pitchers of a single *N.* × *ventrata* plant were used for isolation of total RNA using an RNeasy Plant Mini kit (Qiagen, Valencia, CA, USA). mRNA library preparation and sequencing were performed as described above for *D. rotundifolia*. A total of 23,277,185 read pairs (20,496,137 after filtration) were obtained. RNA-seq read data has been deposited in the NCBI SRA database under accessions SRR8944289-SRR8944300.

Illumina RNA-seq reads were mapped to plastid genomes using HISAT2 v. 2.0.4 [76] with the no-softclip option. To decrease the chance of mapping plastid-like sequences from mitochondrial and nuclear genomes, we filtered out alignments with less than 98% sequence identity using a custom Perl script (available at https://github.com/AVBeletsky/bioinformatics_scripts). A total of 503,433 and 752,633 reads were mapped to plastid genomes of *D. rotundifolia* and *N. × ventrata*, respectively. Single nucleotide polymorphisms (SNPs) were detected using FreeBayes v. 1.2.0 [77].

SNPs with more than 10% of reads supporting a non-reference variant and a minimum 10× mapping depth at the SNP site were retained. 78% and 83% of exon sequences of protein-coding genes of *D. rotundifolia* and *N. × ventrata*, respectively, had at least 10-fold coverage by RNA-seq reads, so some SNPs may be missed. The SNP effect was annotated using SnpEff v. 4.3i [78]. The SNP table obtained was inspected for C to T nucleotide substitutions in protein-coding genes. SNPs with more than three reads supporting the substitution were considered as RNA editing events.

In addition, the PREP-Cp tool (http://prep.unl.edu/) [79] was used to predict RNA editing sites (with a minimal editing score of 0.7).

## Figures and Tables

**Table 1:** Summary of genes identified in the *D. rotundifolia* plastome.

Function	Genes
Photosystem I	*psaA, psaB, psaC, psaI, psaJ, ycf3^i^, ycf4*
Photosystem II	*psbA, psbB, psbC, psbD, psbE, psbF, psbH, psbI, psbJ, psbK, psbL, psbM, psbN, psbT, psbZ*
Cytochrome *b6/f* complex	*petA, petB^i^, petD^i^, petG, petL, petN, ccsA*
ATP synthase	*atpA, atpB, atpE, atpF^i^, atpH, atpI*
RNA polymerase	*rpoA, rpoB, rpoC1^i^, rpoC2*
Ribosomal proteins (large subunit)	*rpl2, rpl14, rpl16^i^, rpl20, rpl22, rpl23, rpl32, rpl33, rpl36*
Ribosomal proteins (small subunit)	*rps2, rps3, rps4, rps7, rps8, rps11, rps12^i^, rps14 (x2), rps15, rps16, rps19*
Other protein-coding genes	*rbcL, infA, matK, cemA, clpP, accD, ycf68*
rRNAs	*rrn16, rrn23, rrn4.5, rrn5*
tRNAs	*trnC-GCA, trnD-GUC, trnE-UUC, trnF-GAA, trnG-GCC, trnH-GUG, trnI-CAU, trnI-GAU^i^, trnK-UUU, trnL-CAA, trnL-UAA^i^, trnL-UAG, trnM-CAU (x2), trnfM-CAU, trnN-GUU, trnP-UGG (x2), trnQ-UUG, trnR-ACG, trnR-UCU, trnS-GCU, trnS-GGA, trnS-UGA, trnT-GGU, trnT-UGU, trnV-GAC, trnW-CCA, trnY-GUA*
Pseudogenes	ψrps18(orf641), ψycf15
Pseudogenes present along with an intact copy	ψinfA, ψpsaB, ψpsaJ, ψpsbI, ψpsbJ, ψrpl2

Genes duplicated in inverted repeats were counted once; *^i^* denotes intron-containing genes, including trans-spliced *rps12*.

**Table 2:** Repetitive sequence content in plastid genomes.

Species	Plastome Size (bp)	Repetitive Sequences (bp)	Repetitive Sequences (%)	Tandem Repeats (bp)
**Caryophyllales; Droseraceae**				
*Drosera rotundifolia*	192,912	32,380	23.13%	736
*Drosera erythrorhiza*	134,391	4708	4.84%	164
*Drosera regia*	136,810	1883	1.66%	175
*Dionaea muscipula*	117,589	5599	4.90%	460
*Aldrovanda vesiculosa*	141,568	2514	2.20%	326
**Caryophyllales; Nepenthaceae**				
*Nepenthes* × *ventrata*	156,637	664	0.51%	82
*Nepenthes mirabilis*	156,381	5426	4.20%	237
**Lamiales; Lentibulariaceae**				
*Pinguicula ehlersiae*	147,147	893	0.74%	151
*Utricularia macrorhiza*	153,228	632	0.50%	476
*Utricularia reniformis*	139,725	682	0.59%	60
*Utricularia gibba*	152,113	780	0.63%	225
*Genlisea margaretae*	141,255	500	0.43%	440
*Genlisea aurea*	140,010	586	0.51%	251
*Genlisea filiformis*	140,308	622	0.54%	204
*Genlisea pygmaea*	140,466	467	0.40%	227
*Genlisea repens*	140,432	467	0.40%	225
*Genlisea tuberosa*	140,677	563	0.49%	208
*Genlisea violacea*	143,416	769	0.65%	60
**Caryophyllales; Caryophyllaceae**				
*Silene noctiflora*	151,639	2852	2.34%	225
*Silene chalcedonica*	148,081	4566	3.67%	135
*Silene conica*	147,208	1785	1.48%	40
*Silene conoidea*	147,896	1615	1.33%	151
*Silene paradoxa*	151,632	2023	1.60%	256
*Silene latifolia*	151,736	1189	0.94%	20
*Silene vulgaris*	151,583	1121	0.89%	181
*Agrostemma githago*	151,733	1297	1.03%	105

Reported repeat content excludes one copy of the large IR.

**Table 3:** RNA-editing pattern in *D. rotundifolia* plastome identified by RNAseq read alignment.

Gene	Amino Acid Position	Codon *	Amino Acid Change	Editing Frequency	PREP-Cp Editing Score
*atpF*	31	CcA	P=>L	98%	0.86
*rps2*	54	AcA	T=>I	72%	0.71
*rps2*	92	TcA	S=>L	93%	1.00
*rps14*	50	CcA	P=>L	67%	1.00
*rpl22*	73	TcA	S=>L	70%	**
*rpl23*	24	TcT	S=>F	81%	0.71

* lower-case ‘c’ indicates the edited position; ** PREP-Cp does not analyze this gene.

## Supplementary Materials

Supplementary Materials can be found at https://www.mdpi.com/1422-0067/20/17/4107/s1. Figure S1: Predicted protein product of orf641. Table S1: Summary of genes identified in the *N.* × *ventrata* plastome. Table S2: RNA-editing pattern in *N.* × *ventrata* plastome predicted by PREP-Cp and identified by RNAseq read alignment. Table S3: RNA-editing pattern in *D. rotundifolia* plastome predicted by PREP-Cp. Table S4: Repeat regions in plastidial rps18 genes. Table S5: Primers used for *D. rotundifolia* plastome assembly.

## Abbreviations

LSC	Large single-copy
SSC	Short single-copy
IR	Inverted repeat
