# Supplementary material for: Plastid Genomes of Carnivorous Plants Drosera rotundifolia and Nepenthes × ventrata Reveal Evolutionary Patterns Resembling Those Observed in Parasitic Plants
Source: Int J Mol Sci. 2019 Aug 22;20(17):4107. doi: 10.3390/ijms20174107 (PMC6747624; doi:10.3390/ijms20174107)
Supplement: Supplementary file 1 [file ijms-20-04107-s001.zip › Supplemental Figure S1.pdf]

Supplemental Figure S1. Predicted protein product of *orf641*

(A) Amino acid sequence of Orf641

```
MDKDKKSPDKDKKSPFSKKVWKK APKQPNI GKKEPNI GPKEPYI GPKEPYA
GQKQLNV GQKQLNV GQKQPNI GQKQPNI GPKEPYV GQKQLNV GQKQPNI
GQKQPNI GPKEPYV GQKQLNV GQKQANI GKKEPNI GPKEPYA GQKQLNV
GQKQLNV GQKQPNI GQKQPNI GPKEPYV GQKQLNV GQKQPNI GQKQPNI
GPKEPYV GQKQLNV GQKQPNI GKKESYV GPKEPYV GQKQLNV GQKQPNI
GKKESYV GPKEPYV GQKQLNV GQKQANI GQKQPNI GQKQPNI GPKEPNI
GKKPPNL GQKQANI GQKQPNI GQKQPNI GQKQPYI GKKESYV GPKEPYV
GQKQLNI GKKQANI SPKQANI GPKQPNI GKKPPNI SPKQPNL GKKPPNL
GPKQPYV GKKQANI GKMKPYI GKMEPYV GKKQPNV GQKQPYI GQKPPYI
GKMESNV GQKQPNV GQKQPNV GQKQPYI GQKQPNV GQKQPNV GQKQPYI
GQKQPNV GQKQPYI GQKSPYI SKKSPYI SKKSPYI SKKSPYI VKKRK
KGKRPFRIRCPQIKSGDQISYRNVRLINKFLSRKTAEIFPRRITKLTFKQQRLLS
SAIKQARILSLLPFFKYPEKRFTKDQKRFKKDTKRFKKYKKRKIQKT
```

N-terminal region containing 73 copies of repeat with GQKQPNI consensus sequence is shown in yellow. Rps18-like region is shown in grey.

(B) Consensus sequence of the repeat

Position	1	2	3	4	5	6	7
Number of occurrences	G-67; S-5; A-1	Q-40; K-17; P-16	K-70; M-3	Q-46; E-18; S-4; P-4; K-1	P-52; L-11; A-6; S-4	N-47; Y-26	I-39; V-29; L-3; A-2

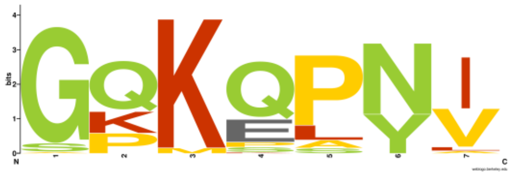

(C) Alignment of amino acid sequences of Rps18-like region of Orf641 of *Drosera rotundifolia* and 86 a.a. long consensus sequence of chloroplast Rps18 domain (CHL00077)

```
Orf641 1    MDKDKKS--KGKRPFRIRCPQIKSGDQISYRNVRLINKFLSRKTAEIFPRRITKLTFKQQR  59
            MDK K+   K KR FR R P I+SGD+I Y+N+ L+++F+S +   +I  RR+T+LT KQQR
       1    MDKSKRPFRKSKRSFRRRLPPIQSGDRIDYKNMSLLSRFIS-EQGKILSRRVTRLTLKQQR  60

Orf641 60   LLSSAIKQARILSLLPFFKYPEKRFTKDQKRFKKDTKRFKKYKKRKIQKT
            L++ AIKQARILSLLPF    EK+F +
       61   LITKAIKQARILSLLPFLNN-EKQFER   86
```

Rps18-like region in Orf641 is shown in grey.
