# Supplementary material for: Plastid Genomes of Carnivorous Plants Drosera rotundifolia and Nepenthes × ventrata Reveal Evolutionary Patterns Resembling Those Observed in Parasitic Plants
Source: Int J Mol Sci. 2019 Aug 22;20(17):4107. doi: 10.3390/ijms20174107 (PMC6747624; doi:10.3390/ijms20174107)
Supplement: Supplementary file 1 [file ijms-20-04107-s001.zip › Supplemental Table S1.doc]

Supplemental Table S1. Summary of genes identified in the *N.* × *ventrata* plastome.

| Function | Genes * |
| --- | --- |
| Photosystem I | *psaA, psaB, psaC, psaI, psaJ, ycf3 ^i^, ycf4* |
| Photosystem II | *psbA, psbB, psbC, psbD, psbE, psbF, psbH, psbI, psbJ, psbK, psbL, psbM, psbN, psbT, psbZ* |
| Cytochrome *b6/f* complex | *petA, petB^i^, petD^i^, petG, petL, petN* |
| ATP synthase | *atpA, atpB, atpE, atpF ^i^, atpH, atpI* |
| NADH dehydrogenase | *ndhA ^i^, ndhB ^i^, ndhC, ndhD, ndhE, ndhF, ndhG, ndhH, ndhI, ndhJ, ndhK* |
| RNA polymerase | *rpoA, rpoB, rpoC1 ^i^, rpoC2* |
| Ribosomal proteins (large subunit) | *rpl2 ^i^,rpl14, rpl16 ^i^, rpl20, rpl22, rpl23, rpl32, rpl33, rpl36* |
| Ribosomal proteins (small subunit) | *rps2, rps3, rps4, rps7, rps8, rps11, rps12 ^i^, rps14, rps15, rps16 ^i^, rps18, rps19* |
| Other protein-coding genes | *rbcL, infA, matK, cemA, clpP ^i^, accD, ycf1, ycf2* |
| rRNAs | *rrn16, rrn23, rrn4.5, rrn5* |
| tRNAs | *trnA-UGC ^i^, trnC-GCA, trnD-GUC, trnE-UUC, trnF-GAA, trnG-GCC,* *trnG-UCC ^i^, trnH-GUG, trnI-CAU, trnI-GAU ^i^, trnK-UUU ^i^, trnL-CAA, trnL-UAA ^i^, trnL-UAG, trnM-CAU, trnfM-CAU, trnN-GUU, trnP-UGG), trnQ-UUG, trnR-ACG, trnR-UCU, trnS-GCU, trnS-GGA, trnS-UGA, trnT-GGU, trnT-UGU, trnV-GAC, trnV-UAC ^i^, trnW-CCA, trnY-GUA* |
| Pseudogenes | ψycf15, ψycf68, ψccsA |
|  |  |

* Genes duplicated in inverted repeats were counted once, *^i^* denotes intron-containing genes, including trans-spliced *rps12*
