# Supplementary material for: Plastid Genomes of Carnivorous Plants Drosera rotundifolia and Nepenthes × ventrata Reveal Evolutionary Patterns Resembling Those Observed in Parasitic Plants
Source: Int J Mol Sci. 2019 Aug 22;20(17):4107. doi: 10.3390/ijms20174107 (PMC6747624; doi:10.3390/ijms20174107)
Supplement: Supplementary file 1 [file ijms-20-04107-s001.zip › Supplemental Table S2.doc]

**Supplemental Table S2**. RNA-editing pattern in *N.* × *ventrata* plastome predicted by PREP-Cp and identified by RNAseq read alignment

| Gene | Amino acid position | Codon* | Amino acid change | PREP-Cp Editing score ** | Confirmed by RNAseq, frequency*** |
| --- | --- | --- | --- | --- | --- |
| *accD* | 65 | cAT | H=>Y | 0.75 | No |
|  | 127 | CcG | P=>L | 0.75 | No |
|  | 238 | cTT | L=>F | 1.00 | No |
|  | 264 | TcG | S=>L | 0.80 | Yes, 87% |
|  | 461 | CсA | P=>L | not found | Yes, 82% |
| *atpA* | 258 | TcA | S=>L | 1.00 | Yes, 84% |
|  | 305 | TcA | S=>L | 1.00 | Yes, 74% |
|  | 424 | cCC | P=>S | 1.00 | No |
|  | 481 | AcA | T=>I | not found | Yes, 62% |
| *atpF* | 31 | CcA | P=>L | 0.86 | Yes, 77% |
| *atpI* | 210 | TcA | S=>L | 1.00 | Yes, 96% |
| *ccsA* | 203 | cTT | L=>F | 0.71 | No |
| *clpP* | 186 | cAT | H=>Y | 1.00 | Yes, 83% |
| *matK* | 24 | cTT | L=>F | 1.00 | No |
|  | 152 | cAT | H=>Y | 1.00 | Yes, 93% |
|  | 214 | cAT | H=>Y | 1.00 | Yes, 69% |
|  | 229 | cCC | P=>S | 1.00 | No |
|  | 237 | TcT | S=>F | not found | Yes, 91% |
|  | 412 | TcT | S=>F | 0.71 | No |
| *ndhA* | 114 | TcA | S=>L | 1.00 | No |
|  | 193 | TcA | S=>L | 1.00 | Yes, 18% |
| *ndhB* | 50 | TcA | S=>L | 1.00 | LC (8/9) |
|  | 156 | CcA | P=>L | 1.00 | LC (3/9) |
|  | 181 | AcG | T=>M | 1.00 | LC (2/4) |
|  | 196 | cAT | H=>Y | 1.00 | Yes, 50% |
|  | 204 | TcA | S=>L | 0.80 | No |
|  | 246 | CcA | P=>L | 1.00 | Yes, 75% |
|  | 249 | TcT | S=>F | 1.00 | Yes, 86% |
|  | 277 | TcA | S=>L | 1.00 | LC (3/6) |
|  | 279 | TcA | S=>L | 1.00 | LC (4/8) |
|  | 494 | CcA | P=>L | 1.00 | Yes, 38% |
| *ndhC* | 108 | TcA | S=>L | NA | Yes, 44% |
| *ndhD* | 123 | TcA | S=>L | 1.00 | Yes, 38% |
|  | 220 | TcA | S=>L | 1.00 | Yes, 65% |
|  | 288 | TcA | S=>L | 1.00 | Yes, 57% |
|  | 291 | CcC | S=>L | 1.00 | Yes, 42% |
|  | 428 | TcA | S=>L | 0.80 | LC (4/8) |
|  | 432 | TcA | S=>L | 0.80 | Yes, 27% |
| *ndhE* | 78 | CcA | P=>L | NA | Yes, 91% |
| *ndhF* | 46 | TcA | S=>L | 0.80 | No |
|  | 97 | TcA | S=>L | 1.00 | LC (4/6) |
|  | 196 | cTT | L=>>F | 0.80 | No |
|  | 502 | TcT | S=>F | 1.00 | No |
|  | 582 | AcA | T=>I | 1.00 | No |
| *ndhG* | 56 | cAT | H=>Y | 0.80 | No |
|  | 105 | AcA | T=>I | 0.80 | No |
| *ndhK* | 22 | TcA | S=>L | NA | Yes, 92% |
| *petB* | 144 | cGG | R=>W | 1.00 | Yes, 93% |
| *petD* | 139 | GcG | A=>V | 0.86 | No |
| *petL* | 2 | CcT | P=>L | not found | Yes, 54% |
| *psaI* | 29 | cAT | H=>Y | not found | Yes, 70% |
| *psbE* | 72 | cCT | P=>S | 1.00 | Yes, 91% |
| *psbF* | 26 | TcT | S=>F | 1.00 | Yes, 89% |
| *psbZ* | 17 | TcA | S=>L | NA | Yes, 33% |
| *rpoA* | 67 | TcT | S=>F | not found | Yes, 49% |
|  | 123 | TcA | S=>L | 1.00 | No |
|  | 281 | cCC | P=>S | 0.71 | No |
| *rpoB* | 113 | TcT | S=>F | 1.00 | LC (6/6) |
|  | 184 | TcA | S=>L | 1.00 | LC (4/4) |
|  | 189 | TcG | S=>L | 1.00 | LC (1/1) |
|  | 797 | TCc | S=>S | not found | Yes, 19% |
|  | 809 | TcA | S=>L | 0.86 | Yes, 60% |
| *rpoC1* | 14 | TcA | S=>L | 1.00 | Yes, 64% |
|  | 153 | CcC | P=>L | not found | Yes, 16% |
|  | 213 | AcT | T=>I | 0.86 | No |
|  | 220 | GcA | A=>V | 0.71 | No |
|  | 347 | CcA | P=>L | not found | Yes, 13% |
|  | 424 | TcG | S=>L | not found | Yes, 12% |
|  | 438 | CcG | P=>L | not found | Yes, 13% |
|  | 664 | cTT | L=>F | 0.71 | No |
| *rpoC2* | 238 | AcG | T=>M | 0.75 | No |
|  | 502 | AcG | T=>M | 0.86 | No |
|  | 533 | cTT | L=>F | 1.00 | No |
|  | 764 | cGG | R=>W | 1.00 | No |
|  | 994 | cAT | H=>Y | not found | Yes, 93% |
| *rps2* | 45 | AcA | T=>I | 0.71 | Yes, 100% |
|  | 83 | TcA | S=>L | 1.00 | Yes, 89% |
| *rps8* | 48 | GcG | A=>V | 1.00 | No |
| *rps12* | 36 | TcA | S=>L | NA | LC (7/9) |
| *rps14* | 27 | TCA | S=>L | 1.00 | Yes, 96% |
|  | 50 | CCA | P=>L | 1.00 | Yes, 87% |
| *rps18* | 74 | TcG | S=>L | NA | Yes, 64% |
| *rpl23* | 24 | TcT | S=>F | 0.71 | Yes, 94% |

* lower-case ‘c’ indicates the edited position

** not found, not predicted by PREP-Cp; NA, this gene is not analyzed by PREP-Cp

*** LC, coverage at the potential editing site <10 (numbers of edited/unedited reads)
