# Supplementary material for: Plastid Genomes of Carnivorous Plants Drosera rotundifolia and Nepenthes × ventrata Reveal Evolutionary Patterns Resembling Those Observed in Parasitic Plants
Source: Int J Mol Sci. 2019 Aug 22;20(17):4107. doi: 10.3390/ijms20174107 (PMC6747624; doi:10.3390/ijms20174107)
Supplement: Supplementary file 1 [file ijms-20-04107-s001.zip › Supplemental Table S3.doc]

**Supplemental Table S3**. RNA-editing pattern in *D. rotundifolia* plastome predicted by PREP-Cp.

| Gene | Amino acid position | Codon* | Amino acid change | PREP-Cp Editing score | Confirmed by RNAseq, frequency |
| --- | --- | --- | --- | --- | --- |
| *accD* | 247 | TcT | S => F | 1.00 | No |
|  | 257 | GcA | A => V | 1.00 | No |
|  | 305 | cAT | H => Y | 0.80 | No |
| *atpA* | 464 | cAT | H => Y | 0.71 | No |
| *atpB* | 170 | AcT | T => I | 1.00 | No |
| *atpF* | 31 | CcA | P => L | 0.86 | Yes, 98% |
| *ccsA* | 21 | GcA | A => V | 1.00 | No |
| *matK* | 349 | TcG | S => L | 0.71 | No |
|  | 422 | TcT | S => F | 0.71 | No |
| *petL* | 2 | CcT | P => L | 0.86 | No |
| *psaB* | 76 | cCA | P => S | 0.71 | No |
| *rpl2* | 87 | cAC | H => Y | 1.00 | No |
| *rol20* | 122 | cCT | P => S | 1.00 | No |
| *rpl23* | 9 | CTT | L => F | 0.86 | No |
|  | 24 | TcT | S => F | 0.71 | Yes, 81% |
| *rpoA* | 265 | AcA | T => I | 0.86 | No |
|  | 279 | cCC | P => S | 0.71 | No |
|  | 318 | AcA | T => I | 0.86 | No |
| *rpoB* | 20 | cTT | L => F | 1.00 | No |
|  | 189 | TcA | S => L | 1.00 | No |
|  | 204 | cTC | L => F | 1.00 | No |
|  | 326 | cCT | P => S | 1.00 | No |
| *rpoC1* | 165 | cTT | L => F | 0.86 | No |
|  | 218 | AcT | T => I | 0.86 | No |
| *rpoC2* | 7 | TcG | S => L | 1.00 | No |
|  | 278 | cTT | L => F | 0.86 | No |
|  | 1113 | cTT | L => F | 0.86 | No |
|  | 1305 | GcT | A => V | 0.71 | No |
| *rps2* | 20 | cTC | L => F | 0.86 | No |
|  | 54 | AcA | T => I | 0.71 | Yes, 72% |
|  | 92 | TcA | S => L | 1.00 | Yes, 93% |
|  | 93 | GcA | A => V | 1.00 | No |
|  | 212 | cCG | P => S | 0.86 | No |
| *rps14* | 27 | TcA | S => L | 1.00 | No |
|  | 34** | GcT | A => V | 0.86 | No |
|  | 50 | CcA | P => L | 1.00 | Yes, 67% |

* lower-case ‘c’ indicates the edited position

** one of two copies of *rps14* already contain GTT codon at the DNA level
