# Supplementary material for: Plastid Genomes of Carnivorous Plants Drosera rotundifolia and Nepenthes × ventrata Reveal Evolutionary Patterns Resembling Those Observed in Parasitic Plants
Source: Int J Mol Sci. 2019 Aug 22;20(17):4107. doi: 10.3390/ijms20174107 (PMC6747624; doi:10.3390/ijms20174107)
Supplement: Supplementary file 1 [file ijms-20-04107-s001.zip › Supplemental Table S5.doc]

**Supplemental Table S5.** Primers used for *D. rotundifolia* plastome assembly

| Name | Sequence (5’-3’) | Localization |
| --- | --- | --- |
| Drot_5_R | TTGAGATTCATAGTTATACCTCCT | 59004-59027 |
| Drot_1_R | TGGGCTAAATCTAGCGGAAATCT | 60998-60976 |
|  |  |  |
| Drot_1_F | AAGGCTTTGACTCTGCTAACGA | 97877-97898 |
| Drot_7_R | ACTCTGCCTACTATACATCAACCA | 98743-98720 |
|  |  |  |
| Drot_7_F | ACTTGTGATATTTCGGCTTGGGA | 106224-106246 |
| Drot_4_F | TCTATCTAAGTGAGTATAGTCGAT | 107621-107598 |
|  |  |  |
| Drot_4_R | TTGTATAGATAGATGCATGATCCAT | 121378-121402 |
| Drot_1_F | AAGGCTTTGACTCTGCTAACGA | 122310-122289 |
|  |  |  |
| Drot_5_F | AGCACTATTCCACCTCTTTGCTA | 174489-174511 |
| Drot_3_F | CTTAGATCCTGCTTTGCCCAA | 175188-175168 |
|  |  |  |
| Drot_3_R | TGATTTGATCTGTGGACAACGTAT | 191032-191055 |
| DrotS1_3R | TTCCATTTTACCTATATAAGGTTTCA | 191437-191462 |
| DrotS1_10F | AGAAAGTTTGGAAGAAAGCACCTA | 192641-192618 |
| Drot_10_F | ACTACCAATGAGTTCTATTCTATAATA | 104-78 |
|  |  |  |
| Drot_10_R | TCTAGCATGACCACTTGATGAAAT | 3408-3431 |
| Drot_2_R | TCTTTCTACCGAGCTAAAACAACAAT | 5606-5581 |
|  |  |  |
| Drot_2_F | AGACCATGTAATTCGCTAAATCCATCA | 31179-31205 |
| Drot_6_R | AGTATCTCGACCTCTAACTACTA | 32004-31982 |
|  |  |  |
| Drot_6_F | TATGCTGGGTCAGATCCAGT | 45126-45145 |
| Drot_5_F | AGCACTATTCCACCTCTTTGCTA | 45698-45676 |

PCR amplifications were performed on Mastercycler Gradient PCR machine (Eppendorf) using 1.25 units GoTaq® DNA Polymerase (Promega) per 50μl amplification reaction in the Green GoTaq® Reaction Buffer supplemented with 0.2mM each dNTP and 1.0μM each primer. Cycling conditions were as follows: 96°C for 5 minutes, followed by 40 cycles of 96°C for 40”, 60” at selected annealing temperature and 72°C for 60”, and a final 5 minute elongation step at 72°C. Optimum annealing temperature for each primer pair was determined using gradient PCR.
